# *MDM2* amplification in rod-shaped chromosomes provides clues to early stages of circularized gene amplification in liposarcoma

**DOI:** 10.1038/s42003-024-06307-1

**Published:** 2024-05-20

**Authors:** Saskia Sydow, Paul Piccinelli, Shamik Mitra, Panagiotis Tsagkozis, Asle Hesla, Camila B. R. De Mattos, Jan Köster, Linda Magnusson, Jenny Nilsson, Adam Ameur, René Wardenaar, Floris Foijer, Diana Spierings, Fredrik Mertens

**Affiliations:** 1 Division of Clinical Genetics, Department of Laboratory Medicine, Lund University, 221 84 Lund, Sweden; 2 Department of Clinical Genetics and Pathology, Office for Medical Services, Region Skåne, 221 85 Lund, Sweden; 3 Department of Orthopedics, Karolinska University Hospital, Solna, 17176 Stockholm, Sweden; 4 Department of Orthopedics, Skåne University Hospital, 221 85 Lund, Sweden; 5 Department of Immunology, Genetics and Pathology, Science for Life Laboratory, Uppsala University, Uppsala, Sweden; 6 European Research Institute for the Biology of Ageing (ERIBA), University of Groningen, University Medical Center Groningen, Groningen, the Netherlands

**Keywords:** Chromosomes, Genomic instability

## Abstract

Well-differentiated liposarcoma (WDLS) displays amplification of genes on chromosome 12 (Chr12) in supernumerary ring or giant marker chromosomes. These structures have been suggested to develop through chromothripsis, followed by circularization and breakage-fusion-bridge (BFB) cycles. To test this hypothesis, we compared WDLSs with Chr12 amplification in rod-shaped chromosomes with WDLSs with rings. Both types of amplicons share the same spectrum of structural variants (SVs), show higher SV frequencies in Chr12 than in co-amplified segments, have SVs that fuse the telomeric ends of co-amplified chromosomes, and lack interspersed deletions. Combined with the finding of cells with transient rod-shaped structures in tumors with ring chromosomes, this suggests a stepwise process starting with the gain of Chr12 material that, after remodeling which does not fit with classical chromothripsis, forms a dicentric structure with other chromosomes. Depending on if and when telomeres from other chromosomes are captured, circularized or linear gain of 12q sequences will predominate.

## Abstract (web summary)

Amplification in liposarcoma develops through a stepwise process that does not fit with classical chromothripsis. Depending on if and when telomeres from other chromosomes are captured, circularized or linear gain of 12q sequences will predominate.

## Introduction

Gene amplification is a common phenomenon in cancer, resulting in overexpression of key oncogenes^1^. From a cytogenetic point of view, three major manifestations of gene amplification have been discerned: double minutes (dmin), homogeneously staining regions (hsr), and ring and giant marker chromosomes (RGMCs)^2–6^. These forms of gene amplification are likely to arise through different mechanisms, but in contrast to the extensive literature on the origin of dmin and hsr, little is still known about how RGMCs arise and develop. Partly, this ignorance could be related to the large size of RGMCs, as well as the frequent involvement of multiple chromosomes in the same amplified structures. In addition, RGMCs are mitotically highly unstable^7,8^ and, in combination with selective forces acting on tumor cells, they have thus undergone extensive remodelling at the time they can be studied in tumors. RGMCs likely correspond to so-called ”neochromosomes” with ”seismic” amplification identified through DNA sequencing studies^9,10^, i.e., large amplicons with multiple levels of copy number (CN) gain. Such amplicons are more common in certain tumor types, notably in a soft tissue tumor known as atypical lipoma/well-differentiated liposarcoma (WDLS). Based on sequencing data, it has been suggested that the initiating event would be a chromothriptic shattering of a chromosomal segment, followed by circularization and further repetitive rounds of circular recombination^9,10^. Chromothripsis is a term introduced in 2011 to describe massive localized shattering, presumably in a single event, of chromosomes^11^. To distinguish chromothripsis from other forms of chromosomal rearrangements, a number of criteria have since been proposed, including that breakpoints should be clustered and give rise to CN levels oscillating between two or three levels; importantly, there should be interspersed loss of segments. Furthermore, restitching of the shattered chromosome parts should give rise to a random rejoining of DNA fragments, i.e., deletion-, duplication-, head-to-head inversion-, and tail-to-tail inversion-type SVs occurring at equal frequencies^12^.

Here, we wanted to assess the validity of the chromothripsis-circularization-BFB model for the origin of amplicons in WDLS and other lipomatous tumors. In order to obtain a better view of the early events in the amplification process, we selected a series of lipomatous tumors in which we previously, using G-banding and fluorescence in situ hybridization (FISH)^13^, had found seemingly stable low-level amplification of sequences from chromosome arm 12q in rod-shaped chromosomes. Assuming that the integration of gained sequences into rod-shaped chromosomes protected the amplicons from further circularized recombination, we employed short-read and long-read whole genome sequencing (shortWGS and longWGS, respectively), genomic arrays (SNP array), single cell whole genome sequencing (scWGS), and mRNA sequencing (RNA-seq) to compare the characteristics of those integrated structures with amplicons in classical ring chromosomes in lipomatous tumors. These analyses revealed intriguing insights into the early steps of supernumerary RGMC formation. Furthermore, they provided molecular clues to the potential evolutionary link between conventional lipomas and WDLS through the gradual development of typical WDLS-associated amplicons.

## Results

### Copy number changes in chromosome 12

**Fig. 1:**
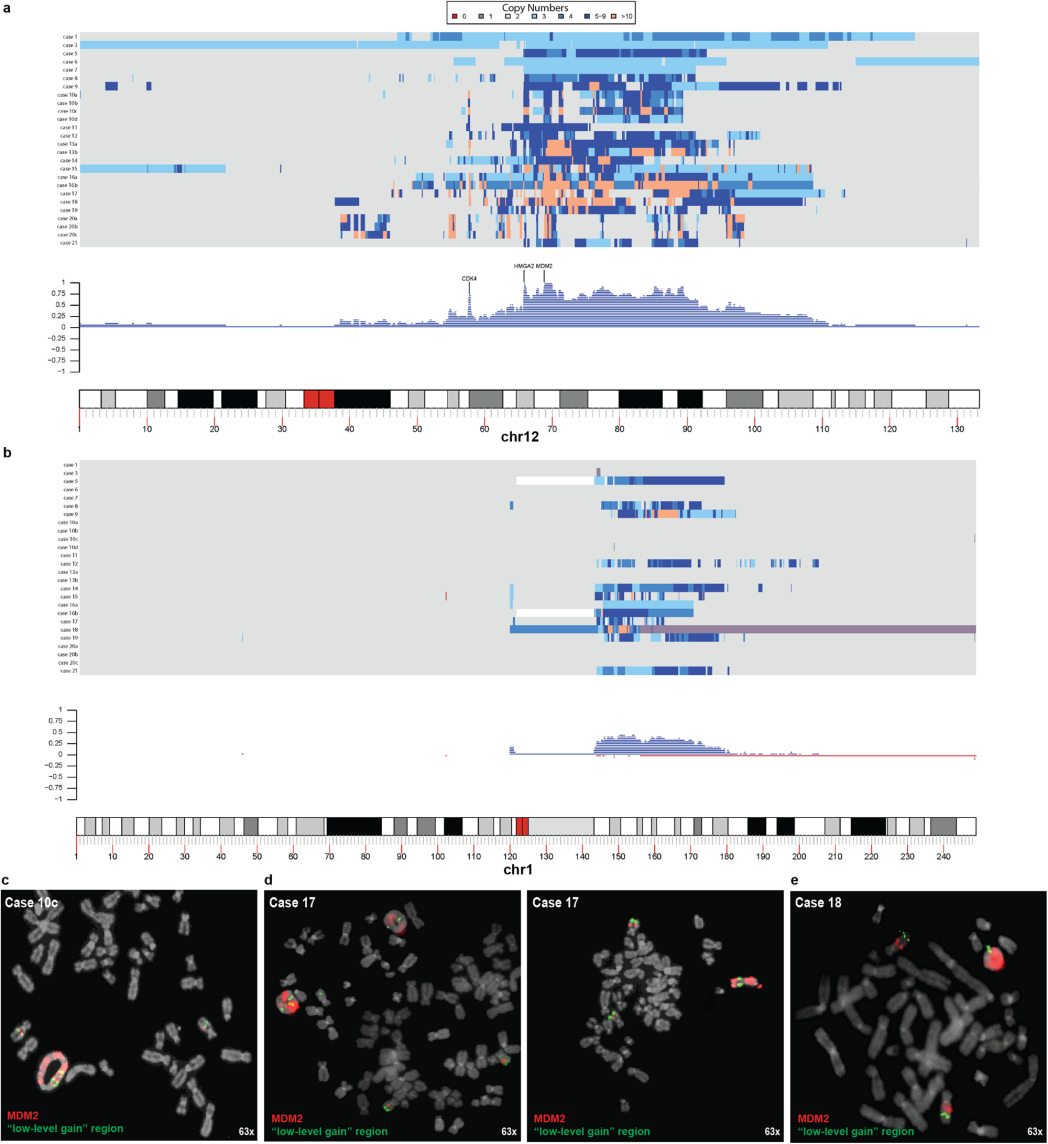
Copy number (CN) variation in lipomatous tumors with 12q gain. Distribution and frequency plots of CN gain in (**a**) Chr12 and (**b**) Chr1 from bulk DNA. Fluorescence in situ hybridization on metaphase spreads from **c** Case 10c, **d** Case 17 and **e** Case 18 shows that also low-level gained segments (green signals; *MDM2* signals in red) are included in the ring chromosomes, as well as in the two normal homologues. Also, Case 17 (**d**) highlights the interchangeability between ring and rod-shaped chromosomes.

A total of 27 tumor samples from 20 patients were analyzed with regard to CN changes, including six with rod-shaped chromosomes (Group A), and 21 with ring chromosomes (Group B). The segmentation of Case 2 failed and could only be analyzed visually. Therefore, Case 2 will not be displayed in any figures. All tumors had CN changes (Supplementary Fig. 1), including gain in Chr12, distributed among 1–23 distinct contigs/cases (Fig. 1a). CN levels ranged from 3–20 at SNP array and from 3–97 at WGS (Supplementary Data 1 and 2). All 21 tumor samples with ring chromosomes (Group B) showed multiple (5–23) CN gained/amplified contigs with multiple CN levels, which is in line with the known mitotic instability of supernumerary ring chromosomes^8,9,14^. As shown here, also WDLS with classical ring chromosomes can have large (>10 Mb) contigs of CN gain, often with long stretches with only one extra copy of Chr12 material (Fig. 1a). In order to verify that such regions with low-level gain were part of ring chromosomes, and not independent secondary events occurring in other chromosomal structures, metaphase FISH was performed with probes mapping to relevant loci, confirming their inclusion in ring chromosomes in 6/7 samples (Fig. 1c–e; Supplementary Fig. 2).

Tumors with CN gain in the form of rod-shaped chromosomes (Group A) differed from and were less complex than those with ring chromosomes (Group B) in several ways (Fig. 1a, b; Supplementary Fig. 1). First, Group A tumors had fewer (median 3 *vs* 10) and longer (median 1,585 Kb *vs* 370 Kb) contigs with CN gain in Chr12 than those in Group B tumors. Second, the total length of gained Chr12 sequences was higher in rod-shaped chromosomes (median 59.4 Mb) than in ring chromosomes (median 26.1 Mb). However, when taking the CN levels into account, tumors with ring chromosomes had more extra Chr12 material than tumors with rod-shaped chromosomes (median 106.8 Mb *vs* median 73.7 Mb). Third, the median CNs for *CDK4* (2 *vs* 9), *HMGA2* (3 *vs* 8), and *MDM2* (3 *vs* 9) were lower in Group A tumors compared to Group B tumors. Fourth, CN transitions were more frequent in ring chromosomes than in rod-shaped chromosomes: the largest gained contig in 12q of each sample showed a median of 1.2 (range 0.37–2.59) CN transitions per Mb in ring chromosomes vs a median of 0 (range 0–0.7) in rod-shaped chromosomes.

**Fig. 2:**
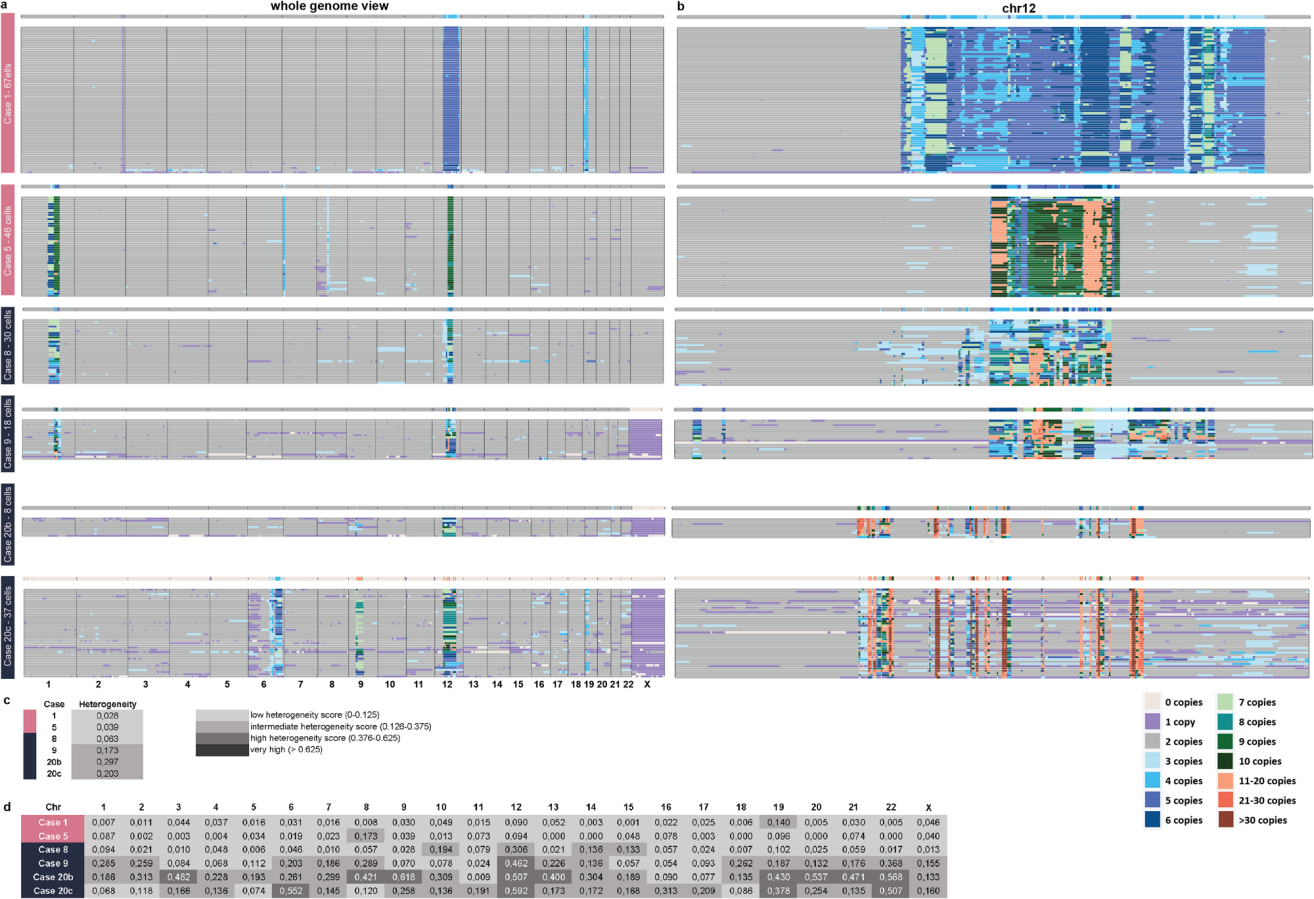
Single cell copy number (CN) variation in lipomatous tumors with 12q gain. Single cell whole genome sequencing with 1 Mb bins showing distribution of (**a**) genome-wide CN changes in individual cells and (**b**) with 40 kb bins showing distribution of CN gain in Chr12 in individual cells. Heterogeneity scores (**c**) genome-wide and (**d**) chromosome-specific based on 1 Mb bins.

To study whether the genomes in tumors with rod-shaped chromosomes were less complex than those in tumors with ring chromosomes also at the single cell level, we performed scWGS on selected tumors (*n* = 2, Group A; *n* = 4, Group B). The distribution of gained and lost sequences in Chr12 at scWGS corresponded well with bulk data from SNP array and shortWGS, but the average CN levels of all analyzed cells were higher in the scWGS profiles (Fig. 2a, b). Furthermore, scWGS revealed extensive intercellular variation in CN levels (Fig. 2a, b). Additionally, the heterogeneity scores for both Chr12 and the entire genome gradually increased from Case 1 to Case 20, with the lowest values in the two cases with rod-shaped chromosomes and one with ring chromosomes (Fig. 2c, d). SVs were largely restricted to chromosomes with CN gain in all cases.

Thus, scWGS confirmed that tumors with Chr12 gain in rod-shaped chromosomes were genomically relatively stable whereas tumors with ring chromosomes demonstrated varying levels of genetic instability. Despite the extensive variation seen at the single-cell level in cases with ring chromosomes, there was only slight variation with time at the bulk DNA level. From three tumors in Group B (Cases 10, 13, and 20), also 1-2 relapse samples were analyzed, with time intervals ranging from 4–12 years between the first and last samples (Table 1; Supplementary Data 1). There was no trend suggesting that gained sequences in Chr1 or Chr12 change in any consistent way regarding their extension or amplitude or that they accumulate more CN transitions/Mb on Chr12 with time (Fig. 1a, b; Supplementary Data 1 and 2).

**Table 1:** Summary of tumor cohort and analyses performed

Case No.	Diagnosis^a^	Ploidy^b^	G-band^c^	SNP array^d^	WGS^e^	RNA-seq^f^	scWGS^g^	Chr12^h^	CN *MDM2*^i^	CN chr12^j^	CN chr1^k^
**Group A: Tumors with gain of MDM2 in rod-shaped chromosomes**											
1	WDLS	Dip	Yes	Yes	shortWGS	Yes	67	1	3/4 (WGS)	3–5/3–7 (WGS)	No
2	WDLS	Dip	Yes	ND	shortWGS	Yes	ND	1	3 (WGS,VI)	3 (WGS, VI)	No
3	Lipoma	Dip	Yes	Yes	ND	Yes	ND	2	3	3	No
4	WDLS	Dip	Yes	ND	ND	Yes	ND	1	5 (FISH)	Unknown	Unknown
5	WDLS	Dip	Yes	ND	shortWGS, longWGS	Yes	46	2	6 (WGS)/7 (FISH)	3–6 (WGS)	3–8 (WGS)
6	Lipoma	Dip	Yes	Yes	ND	Yes	ND	1	3/4 (FISH)	3	No
7	WDLS	Dip	Yes	Yes	shortWGS	Yes	ND	1	3/3 (WGS;FISH)	3/3 (WGS)	No
**Group B1: Tumors with gain of MDM2 in ring chromosomes and with large contigues of 12q gain** > **10** **Mb**											
8	WDLS	Dip	ND (FISH)	Yes	ND	Yes	30	2 (FISH)	4	3–8	3–7
9	WDLS^l^	Dip	ND (FISH)	Yes	ND	Yes	18	2 (FISH)	6	3–15	3–10
10a	WDLS	Dip	Yes	Yes	ND	ND	ND	2	8	3–12	No
10b	WDLS	Dip/Tet	Yes	Yes	ND	ND	ND	2/4	8	3–11	No
10c	WDLS	Dip	Yes	Yes	ND	ND	ND	2	8–10	3–14	No
10d	WDLS	Dip	ND	Yes	ND	Yes	ND	ND	6	3–9	No
11	WDLS	Dip	Yes	Yes	ND	Yes	ND	2	8	5–9	No
12	Lipoma	Dip	Yes	Yes	ND	Yes	ND	2	7	3–7	3–8
13a	WDLS	Dip	Yes	Yes	ND	Yes	ND	2	9	3–11	No
13b	WLDS	Dip	ND	Yes	ND	ND	ND	ND	9	3–16	No
14	WLDS	Dip	ND (FISH)	Yes	ND	Yes	ND	2	8	3–11	3–9
15	WDLS	Dip/Tet	Yes	Yes	ND	ND	ND	2/4	10	3–16	3–13
16a	WDLS	Dip/Tet	Yes	Yes	ND	ND	ND	2/4	12	3–14	3
16b	WDLS	Dip/Tet	ND	ND	shortWGS	Yes	ND	ND	60 (WGS)	4–97 (WGS)	4–13 (WGS)
17	WDLS	Dip	Yes	Yes	ND	Yes	ND	2	10	3–14	3–8
18	WDLS	Dip	ND (FISH)	Yes	ND	Yes	ND	2	19	5–20	1–20
19	WLDS	Dip/Tet	Yes	Yes	ND	Yes	ND	2/4	9	3–11	3–9
**Group B2: Tumors with gain of MDM2 in ring chromosomes and with shorter contigues of 12q gain** < **6** **Mb**											
20a	WDLS	Dip	Yes	Yes	ND	ND	ND	2	11	3–13	No
20b	WDLS	Dip	Yes	Yes	ND	Yes	8	2	13	3–14	No
20c	DDLS	Dip	Yes	Yes	shortWGS	ND	37	2	2–16/36 (WGS)	3–16/3–52 (WGS)	No
21	WDLS	Dip	Yes	Yes	longWGS	Yes	ND	2	8	3–8	3–7

^a^*WDLS* atypical lipoma/well-differentiated liposarcoma, *DDLS* dedifferentiated liposarcoma.

^b^*Dip* diploid, *Tet* tetraploid.

^c^Karyotype from G-banding analysis available; *ND* not done, (*FISH)* ring chromosome confirmed by metaphase FISH.

^d^*SNP* array single nucleotide polymorphism array; *ND* not done.

^e^*WGS* whole genome sequencing, *ND* not done, *shortWGS* short read WGS with x90 coverage, *longWGS* long read WGS.

^f^*RNA-seq* mRNA sequencing, *ND* not done.

^g^*scWGS* single cell whole genome sequencing, No. of cells analyzed shown; *ND* not done.

^h^*Chr12* number of intact chromosomes 12, *ND* not done.

^i^MDM2 number of *MDM2* copies, as estimated with SNP array and/or other method (other method shown in parentheses), *VI* visual inspection.

^j^*CN Chr12* Copy number levels in gained segments on chromosome 12, as estimated with SNP array and/or other method (other method shown in parentheses), *VI* visual inspection.

^k^*CN Chr1* Copy number levels in gained segments on chromosome 1, as estimated with SNP array and/or other method (other method shown in parentheses).

^l^WDLS with minimal atypia (Supplementary Fig. 4).

### Copy number changes in chromosome 1

Almost all WDLS display genomic segments that are co-amplified with Chr12, the most common being proximal 1q^15^. In the present study, a CN increase (ranging from 3–20 copies) affecting 1q was seen in 12 samples from 11 patients, including one Group A tumor (Table 1; Fig. 1b; Supplementary Data 1 and 2; Supplementary Fig. 1). Telomeric sequences from Chr1 were never gained/amplified. The highest CN levels in 1q were similar to those in 12q, except for Case 16 which had only 3 copies of proximal 1q but up to 14 copies from 12q (Fig. 1a and b; Supplementary Data 1 and 2). The most proximal position of gained/amplified segments in 1q cannot with certainty be determined, as the large pericentromeric heterochromatic segment (nt positions ~120–143 Mb) lacks informative probes/sequences. Bearing these caveats in mind, the position of the first CN gain in all cases mapped to proximal 1q (nt positions ~143–147 Mb) and the last amplicon to nt positions ~160–206 Mb (Supplementary Data 1 and 2). As for 12q, the gained sequences in 1q affected several Mb, ranging from 10.7–37.3 Mb in Group B tumors and 34.9 Mb in the single Group A tumor. The median number of CN transitions in the largest gained contigs in 1q was identical to that for 12q in Group B tumors (median 1.1/Mb); the single Group A tumor had 0.46 transitions per Mb (Supplementary Data 1).

In summary, co-amplified sequences in 1q were similar to those in 12q with regard to CN levels and CN dynamics, suggesting a simultaneous amplification. However, the distinctly lower CN level in one case indicated a stepwise co-amplification, prompting further analyses with regard to structural variants (SVs).

### Structural variants

**Fig. 3:**
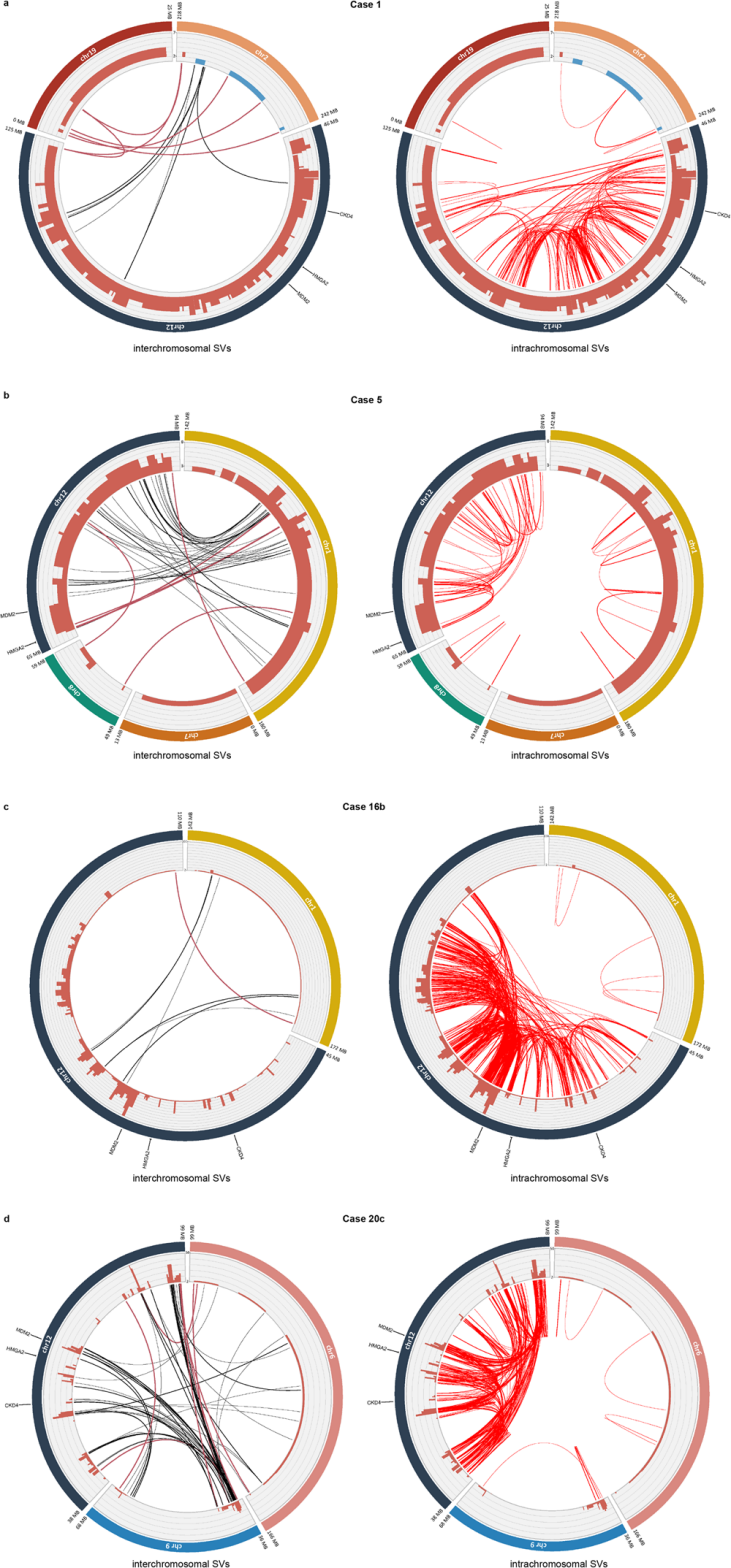
Structural variants in lipomatous tumors with 12q gain. **a** Case 1; **b** Case 5; **c** Case 16b; **d** Case 20c. Inter- (black lines) and intrachromosomal (red lines) structural variants (SVs) detected at whole-genome sequencing in lipomatous tumors with 12q-gain. Copy number state is displayed in terracotta red bars. Important interchromosomal SVs are highlighted in terracotta red.

ShortWGS on three cases from Group A and two from Group B identified a total of 1,117 SVs with an average of 223 SVs per case; >95% of SVs were located in CN altered regions (Fig. 3; Supplementary Data 3). Note that Case 2 could not be analyzed regarding SVs, due to lack of segmentation data. Furthermore, 24–30% of the SVs had at least one breakpoint located within 10 Kb of a CN transition. The number of SVs was lower in Group A (5–201) than in Group B (351 and 445) (Supplementary Data 3). Apart from Case 7, which had an intrachromosomal duplication of part of 12q as the sole change, all other cases had CN changes in additional chromosomes with SVs supporting a joining of these CN-affected regions (Fig. 3). The frequency of rearrangements within CN-affected segments (i.e., intrachromosomal SVs) varied considerably among cases and among segments. Consistently though, gained segments on 12q showed higher numbers of internal SVs per Mb than co-amplified chromosomes (Fig. 3). SVs/Mb in the amplified region of 12q ranged from 1.85 SVs/Mb in Case 5 to 13.5 SVs/Mb in Case 20, compared to 0.29 SVs/Mb and 0.18 SVs/Mb in 1q in Cases 5 and 16, and 0.1 SVs/Mb and 0.63 SVs/Mb for Chr6 and Chr9, respectively, in Case 20. The main types of intrachromosomal SVs in CN gained segments of 12q in shortWGS data from samples 1, 5, 16b, and 20c were rather equally represented, with 246 tail-to-head (+-) deletions, 215 head-to-tail (-+) duplications, 220 head-to-head (--)inversions, and 219 tail-to-tail (++) inversions (Supplementary Data 3). The same distribution was seen for the 253 SVs with at least one breakpoint mapping within 10 Kb of a CN transition (each SV type accounted for 24–26% of these SVs; Supplementary Data 3).

Because of the lack of matching normal tissue, the analysis of SVs obtained from longWGS in Cases 5 and 21 was focused on SVs in CN-affected regions classified as break-end (BND) events, which constituted 83% (45/54) and 81% (48/59), respectively, of all BNDs. BNDs correspond to translocations and large inversions. For Case 5, 24 BNDs were translocations between Chrs 1 and 12, and intrachromosomal BNDs were more frequent in Chr12 than in Chr1 (0.52 BNDs/Mb vs 0.17 BNDs/Mb) (Supplementary Fig. 3; Supplementary Data 4). A similar trend was seen for Case 21, which had 18 translocations between Chr1 and Chr12, and 1.04 intrachromosomal BNDs/Mb in 12q and 0.35 intrachromosomal BNDs/Mb in 1q (Supplementary Fig. 3; Supplementary Data 4).

In summary, by combining CN and SV data, we could show that the mechanisms behind the gain of extra copies of 12q material vary considerably.

### Mechanisms behind gene amplification (Fig. 4)

**Fig. 4:**
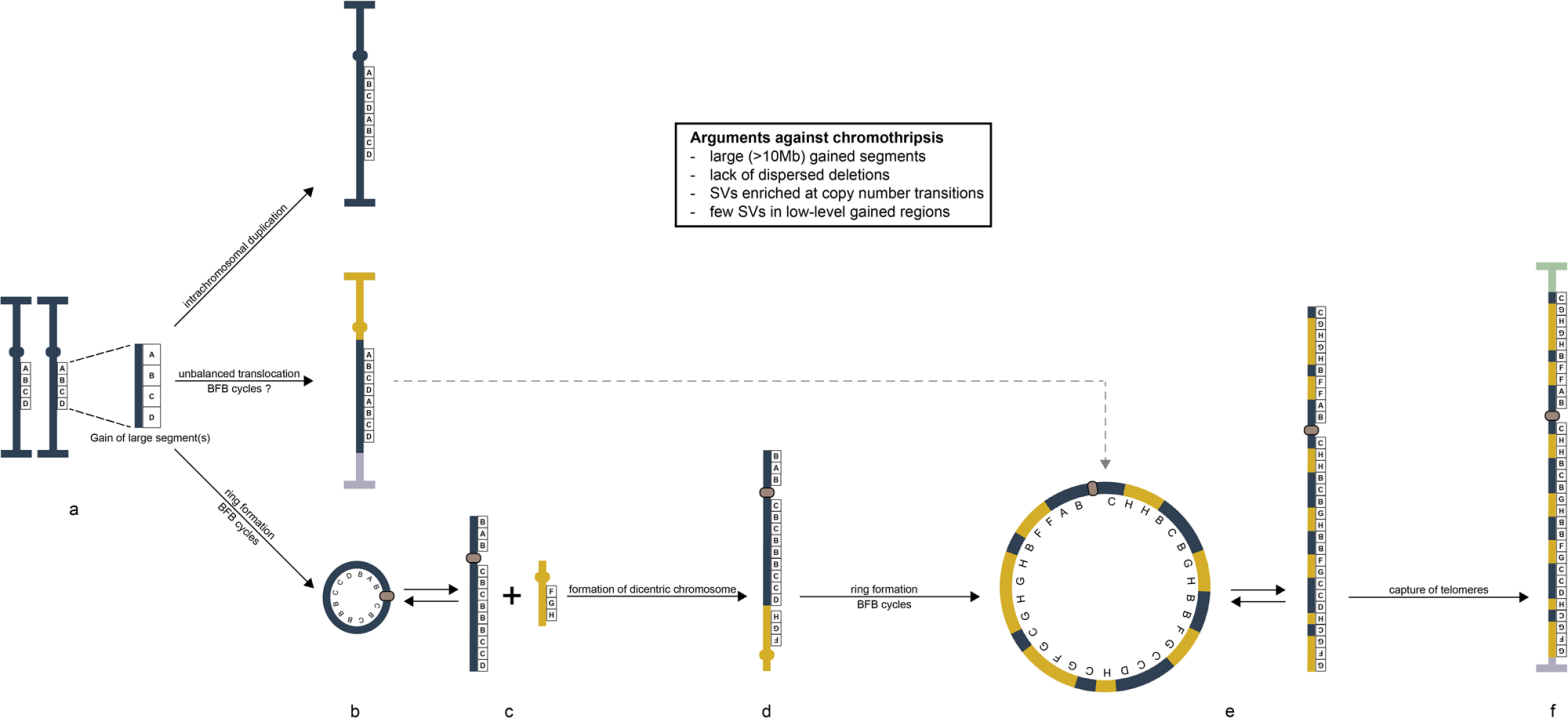
A schematic view of the mechanisms behind gain and amplification of *MDM2* in lipomatous tumors. **a** The initial event is duplication of one or more large (often >10 Mb) segments from chromosome arm 12q (oval circles represent centromeres; horizontal bars represent telomeres). **b** These duplicated segments can, after more or less extensive reorganization, either be (top) inserted in one of the two homologs of Chr12, as exemplified by Case 2, or (middle) translocated to one or more other chromosomes, as exemplified by Case 3; in both scenarios the gained 12q segments end up in a chromosome with intact telomeres, providing relative mitotic stability. The third option (bottom) involves circularization of the gained segments in a ring chromosome, which subsequently develops a neocentromere (beige oval circle); the structural variants, as well as the copy number data in Case 16, suggest that gain of 12q precedes co-amplification with other chromosomes. Due to mitotic instability, the ring chromosome will experience extensive copy number alterations, resulting in amplification of some segments. **c** The ring will occasionally break up to form a rod-shaped structure (see Fig. 1d) and fuse with material from other chromosomes, notably Chr1. **d** The consistent finding of structural variants fusing the most telomeric sequences of the gained sequences from Chr12 and co-amplified chromosomes, as seen in Cases 5, 16, 20, and 21, strongly suggests that the newly formed rod-shaped chromosome is dicentric. **e** After breakage-fusion-bridge cycles, the dicentric chromosome will break and form a new ring chromosome. **f** After further cycles of breaking up and recircularization, the amplified structure will eventually capture telomeres from other chromosomes and become relatively stable.

In the simplest scenario (Fig. 4, top), a gain of 12q-material was achieved through an intrachromosomal duplication of a large (25.5 Mb) segment accompanied by an inversion within the duplicated segment (Case 7). Also, in Cases 2, 3, and 6, the 12q gains could be explained by intrachromosomal duplications; however, they were here accompanied by the separation of gained segments into multiple contigs and, in Case 3 (Fig. 4, middle), translocation with another chromosome. The large size of the segments and the lack of CN loss in them strongly argue against a chromothriptic origin of gained segments in tumors with amplification in rod-shaped chromosomes.

An additional level of complexity was seen in Case 1. G-banding revealed a derivative Chr12 with an internal duplication and addition of unknown material, as well as a derivative Chr19 with 12q material added to 19p; these chromosomes were stable during cell culture^13^. In contrast to the cases above, 12q displayed multiple CN levels and numerous CN transitions (Fig. 1a; Supplementary Data 2). ShortWGS showed that 87% of the SVs were internal 12q rearrangements, strongly suggesting a gain of 12q material as the first event (Fig. 3a; Supplementary Data 3). The relatively equal distribution of different types of SVs in these contigs would fit with chromothripsis, but the lack of interspersed segments with 2 copies, i.e., corresponding to deletions in the supernumerary structure, would not (Supplementary Data 2 and 3). Furthermore, SVs near (≤10 kb) CN transitions were enriched (19% of breakpoints and 29% of SVs *vs* the expected 1.6% and 3.2%, respectively; Supplementary Data 3), strongly suggesting that they were associated with BFB events. The secondary nature of the rearrangements with Chr2 and Chr19 was supported by low numbers of internal SVs and CN levels/transitions in these chromosomes (Fig. 3a; Supplementary Data 2 and 3). Possibly, the supernumerary material from Chr12 could have circularized at an early stage, which would explain the CN variation and part of the internal SVs, and then transformed into rod-shaped chromosomes after translocation with Chrs 2 and 19. Notably, the two most telomeric SVs in the CN-gained parts of Chr12 were translocations with Chr2 and Chr19, respectively, suggesting that the order of contigs in the linear supernumerary Chr12 structure that recombined with Chr2 and Chr19 was similar to that in a normal Chr12.

A further step towards the CN complexity seen in WDLS with classical ring chromosomes (Fig. 4, bottom) was found in Case 5. The tumor cells had two normal copies of Chr12 and the amplified 12q material was present in a derivative Chr8 as well as in a marker chromosome with Chr12 material on both sides of its centromere. Despite the stability of the two chromosomes harboring amplified 12q during cell culture^13^, the array profile revealed extensive CN variation (3–6 copies) and numerous CN transitions (0.70/Mb) (Fig. 1a). Additionally, this case displayed CN gain in proximal 1q, the most commonly co-amplified segment in WDLS^15^, with CN variations (3–8 copies) and CN transitions (0.46/Mb in the largest contig) that were similar to those for 12q (Fig. 1b; Supplementary Data 1 and 2). In line with these findings, FISH revealed that the gained 1q- and 12q-sequences were co-localized, and that the rearranged chromosomes had become rod-shaped by capturing telomeres first from Chr7 and then from Chr8, both of which showed low-level CN changes (Supplementary Fig. 2a and b).

ShortWGS, longWGS and scWGS data confirmed the CN heterogeneity within 1q and 12q amplicons and identified numerous SVs between 1q and 12q (Figs. 2 and 3b; Supplementary Fig. 3a). More frequent internal SVs in CN-gained regions of Chr12 suggest that the gain of Chr12 preceded that of Chr1 (Fig. 3b). However, the CN levels were almost identical, and both SVs and FISH signals showed an intermixed distribution of sequences, arguing for concomitant gain. Furthermore, there was a SV affecting the most distal (telomeric) parts of the CN-gained regions of Chr1 and Chr12, strongly suggesting the formation of a dicentric structure during clonal evolution (Fig. 3b; Supplementary Fig. 3a). Presumably, this was followed by one or more BFB cycles, resulting in circularization of the supernumerary structure, and later integration into rod-shaped chromosomes with help of telomeres from Chr7 and Chr8. Additionally, the evidence for an initial chromothriptic event was weak also in this case; there were no interspersed deletions in Chr12 and SVs were enriched near CN transitions (Supplementary Data 2 and 3).

CN-gained 12q sequences in ring chromosomes (Group B tumors) shared several features with those in rod-shaped chromosomes, including the distribution of SV types, the enrichment of SVs near CN transitions (Fig. 3c and d; Supplementary Fig. 3b; Supplementary Data 3), and the lack of interspersed deletions (Supplementary Data 2). Thus, our results suggest that neither amplicons in rod-shaped chromosomes nor in ring chromosomes originate through a classical chromothriptic mechanism. A schematic view of the potential outcomes of CN gain in 12q, and the dynamic switch between a rod-shaped and a circularized configuration can be seen in Figs. 4 and 1d, respectively.

### Gene expression and fusion genes

**Fig. 5:**
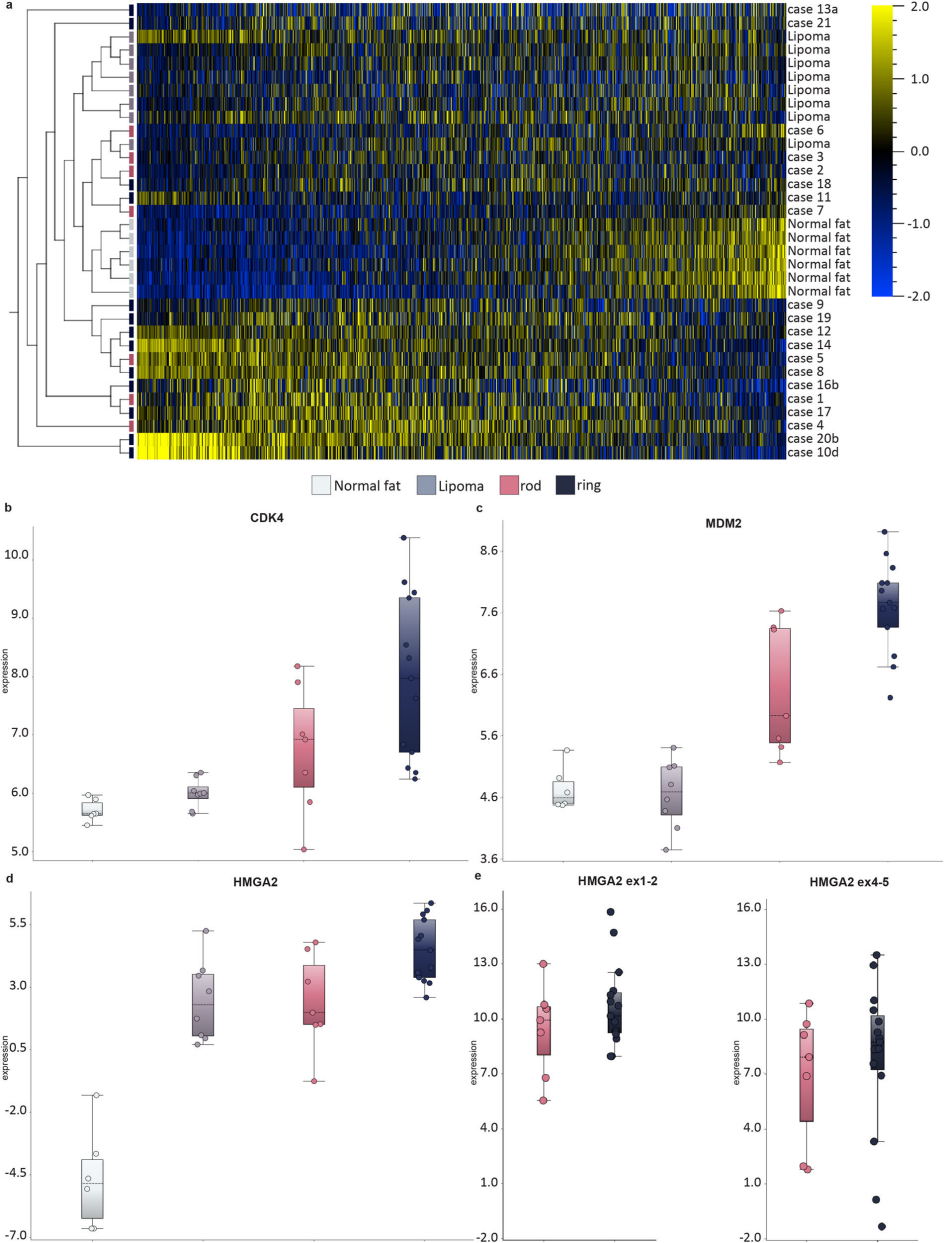
Gene expression profiles in lipomatous tumors with 12q gain at RNA-sequencing (RNA-seq) and quantitative real-time PCR (qRT-PCR). **a** Unsupervised heatmap showing clustering of cases from Group A and B compared to control tissue/tumors (normal fat/lipoma). Box plots at RNA-seq showing expression levels of (**b**) *CDK4*, **c** *MDM2*, **d** *HMGA2*, and at qRT-PCR (**e**, left) *HMGA2* ex1-2; (**e**, right) *HMGA2* ex4-5. Normal fat (*n* = 6), Lipoma (*n* = 8), rod-shaped (*n* = 7, Cases 1–7), ring (*n* = 13, Cases 8–21).

RNA-seq was performed on 20 tumors with extra 12q-material in rod-shaped chromosomes (*n* = 7; Group A) or ring chromosomes (*n* = 13; Group B) (Table 1; Supplementary Data 1). The gene expression profiles were compared with those in eight *HMGA2* fusion-positive conventional lipomas without 12q-gain and six normal fat samples. The unsupervised heatmap (SD: 0.345; 1,109 genes) showed a sub-clustering of lipomas and normal fat (Fig. 5a). However, four samples from Group A and four from Group B blended into the *HMGA2* fusion-positive lipomas. The remaining three samples from Group A and nine from Group B clustered together in a separate block. No clear separation between Groups A and B was seen (Fig. 5a). Focusing on the expression of *CDK4, HMGA2* and *MDM2*, a gradual increase of expression could be observed for *CDK4* and *MDM2* (Fig. 5b and c), with Group B tumors showing the highest median expression levels. Although the variation was extensive in Groups A and B, the median expression levels of *MDM2* were significantly higher in Group A than in lipomas (fold change (fc): 3.22; *P* = 0.0007), and significantly higher in Group B than in Group A (fc: 2.9; *P* = 0.002); the increase in *CDK4* expression was not significant (*P* > 0.05). The expression levels of *HMGA2* were similar in lipomas and Group A tumors, but Group B tumors had higher expression than Group A tumors (fc: 4.23; *p*-value: 0.05; Fig. 4d). Because the RNA-seq data on *HMGA2* expression were based on expression levels of all exons, tumors with *HMGA2* rearrangements could have misleading values. We thus performed qRT-PCR for the 5’- and 3’-ends of *HMGA2* in 22 samples from 20 patients (Fig. 5e; Supplementary Fig. 4). The average log_2_ values were in agreement with RNA seq data, and the expression of the 5’-end of *HMGA2* was slightly higher in Group B samples than in Group A samples, but the difference was not significant (*P* > 0.05; Fig. 5e).

A total of 4,885 fusion transcripts were reported by FusionCatcher. After filtering, 24 (0–4 fusion transcripts per case) remained. None of the fusion transcripts was recurrent (Supplementary Data 5).

In summary, there is no clear-cut transcriptomic border between lipoma and WDLS or between tumors with gain/amplification in rod-shaped chromosomes or ring chromosomes.

## Discussion

Although the concept of gene amplification in neoplasia is of long-standing, deep sequencing has dramatically improved the possibilities to study the architecture and evolution of amplicons. Using WGS data on >2500 cancers, it was recently suggested that so-called seismic amplification in so-called neochromosomes are frequent features (~10%) across a variety of cancer types^10^. One tumor type in which these two phenomena were particularly frequent was WDLS. This is in good agreement with previous cytogenetic and molecular genetic data. These tumors consistently display supernumerary structures, typically ring chromosomes, in which a large number of genes (notably *MDM2*) mapping to chromosome arm 12q become amplified and over-expressed^9,16–18^. Indeed, *MDM2* analysis is often used for the diagnosis of WDLS^19,20^.

It is well known that there can be a wide morphologic spectrum within and between cases of WDLS, with some having a lipoma-like phenotype^19,21^. Vice versa, a subset of lipomas with gain and/or low-level amplification of 12q sequences have been found to display “minimal nuclear atypia”^22^. This morphological overlap between lipoma and WDLS was demonstrated in the present study, where five of the cases with rod-shaped chromosomes were diagnosed as WDLS, and two cases with ring chromosomes as lipoma or WDLS with minimal atypia, respectively (Table 1; Supplementary Data 1). Nor is there, as shown here, any clear-cut border between lipoma and WDLS with regard to *HMGA2* status or *MDM2* CN level or expression levels of these two genes, providing molecular support for the existence of an intermediary tumor type. Indeed, the lipomatous tumors with low-level CN gain in 12q fell in between conventional lipomas and WDLS with classical ring chromosomes (Supplementary Fig. 5). Whether such tumors should be called lipoma with minimal atypia or WDLS with minimal atypia remains to be determined. We would favor the use of the latter in cases with signs of complex rearrangements of 12q material. Reasonably, any such tumor, even if the amplified material is integrated in rod-shaped chromosomes, should carry the potential to progress through re-circularization of the amplified sequences. Thus, our data support the hypothesis that a “lipoma” might transform into a WDLS^13,20,22–25^, but we believe this to be an exceedingly rare event.

The dynamics of ring chromosome development in WDLS and other cancers are still poorly understood, but it has been repeatedly claimed that the initial event is chromothripsis involving one or more chromosomes followed by circularization and repetitive breakage-fusion-bridge (BFB) cycles^5,9,10,26^. The latter events, however, blur the initial events by introducing further SVs and CN shifts. Thus, the present study was based on the assumption that extra copies of Chr12 material integrated into rod-shaped chromosomes could be shielded from progressive instability and hence shed light on the early steps in amplicon formation in liposarcomas.

Neither the cases with rod-shaped chromosomes nor the ones with rings fulfilled the criteria for chromothripsis. How, then, do the amplicons in lipomatous tumors arise? Tumors with co-amplification of other chromosomal segments than 12q provided some interesting clues to this question. The consistently higher number of SVs/Mb in CN-gained material from 12q than in co-amplified sequences and the considerably lower levels of CN gain in 1q than in 12q in one tumor (Case 16) strongly suggest a stepwise process starting with a gain of 12q. Additionally, all four WGS analyzed tumors with co-amplification of 1q or 9p and 12q had SVs supporting translocations between the most distal (telomeric) sequences of the respective gained regions. This strongly suggests the formation of a dicentric chromosome, followed by breaks near the centromere in Chr1 or Chr9 and at variable positions in 12q; indeed, dicentric chromosomes with non-telomeric breakpoints have been shown to preferentially break at pericentromeric regions^27^. Finally, also in the rod-shaped marker chromosome in Case 1, the most distal part of the CN gained region of Chr12 was joined with other chromosomes.

Thus, our data suggest that *MDM2* amplification in lipomatous tumors starts with a gain of one or more large segments of Chr12. Sometimes, the CN gain remains intrachromosomal as a relatively stable structure. Often, however, the initial CN gain occurs as a supernumerary structure, which may oscillate between a circularized and a rod-shaped structure. Depending on at which stage this rod-shaped structure recombines with other chromosomes and whether they contribute telomeric sequences or not, circularized or rod-shaped amplicons will predominate (Fig. 4). An interchangeability between circularized and linear forms of amplified 12q sequences is already known from cytogenetic data; one-fourth of the 185 published karyotypes from WDLS cases have shown clonal giant marker chromosomes, typically together with supernumerary ring chromosomes^28^. As shown here, it is not only the clonal, stabilized giant marker chromosomes with telomeres from other chromosomes that occur, but also seemingly transient rod-shaped structures (Supplementary Fig. 2), which could form dicentrics with other chromosomes and, after further BFB cycles, circularize again. The occurrence of transient rod-shaped chromosomes would also explain why some local recurrences display co-amplified sequences that are not found in the primary tumor^14^.

In summary, we could show that the CN gained Chr12 segments, irrespective of whether they were present in ring chromosomes or in rod-shaped chromosomes, deviated from the criteria for chromothripsis, as defined by Korbel and Campbell (2013)^12^. The amplicons in WDLS seem to develop through a different mechanism, typically involving breakage of supernumerary DNA sequences gained after DNA synthesis, followed by recombination with other chromosomes as rod-shaped structures, and further gene amplification as ring chromosomes. However, why the amplification usually occurs after DNA synthesis in WDLS, and why the end result usually is one or more large ring chromosomes rather than smaller hsrs is unclear. The large size of the amplicons and the frequent co-amplification of *MDM2* with many other genes (e.g., *CDK4*, *HMGA2*) strongly suggest the need for cooperative effects. Indeed, it has been experimentally shown that the concomitant overexpression of multiple genes is critical for WDLS tumorigenesis^29^; such co-amplification of genes/segments from distinct parts of one or more chromosomes is possible in ring chromosomes, but difficult to achieve in the relatively small amplicons found in dmin or hsr.

## Materials and methods

### Tumors

The study included 28 tumor samples with gain or amplification of *MDM2* from 21 patients with lipomatous tumors (Table 1; Supplementary Data 1). The samples included in the present study were selected from a series of >300 deep-seated lipomatous tumors studied by cytogenetics or SNP array during a period of >30 years. First, we included all cases (Cases 1–7) with rod-shaped chromosomes detected at G-banding analysis and from which material was available for molecular studies; two were diagnosed as conventional lipoma and five as WDLS; this set of tumors is hereafter referred to as Group A. To evaluate how such structures differ from amplification in traditional ring chromosomes, we assessed copy number profiles from SNP array analysis of 65 other lipomatous tumors with extra copies of the *MDM2* gene (unpublished data). In 12 of them (one diagnosed as conventional lipoma and eleven as WDLS; Cases 8–19; Group B1), all of which had ring chromosomes, we found at least one uninterrupted contig of copy number gain ≥10 Mb (range 10–49 Mb), making them relevant for comparison with samples in Group A. Finally, two WDLS (Cases 20 and 21) with ring chromosomes harboring only shorter (<6 Mb) amplified contigs of 12q, were included for comparison (Group B2). Clinical information (up-dated, when possible, in the present study) and cytogenetic, FISH, and/or quantitative RT-PCR (qRT-PCR) data for Cases 1, 2, 4–6, 10a-c, 11, 12, 15, 17, 20a, and 21 have been partly presented before^13,14,30–33^. All tumors were deep-seated and classified according to WHO criteria^19,34^. Multiple (2–4 per case) samples could be analyzed from four of the patients (Cases 10, 13, 16 and 20).

Samples were obtained after informed consent from the patients or their legal guardians and ethical approval was obtained from the Swedish Ethical Review Authority (EPN 2017/796). All ethical regulations relevant to human research participants were followed.

### Global bulk DNA copy number profiling

All 21 cases except Case 4 were analyzed with regard to global CN status using DNA extracted from fresh or fresh frozen tumor samples (Table 1; Supplementary Data 1). In 24 samples from 18 patients, the CN profile was obtained through Affymetrix Cytoscan HD SNP arrays (Affymetrix, Santa Clara, USA), as described^35^. In seven samples from seven patients, a CN profile was obtained using shortWGS data; SNP array data were used for CN estimates in three cases that were analyzed with both SNP array and shortWGS. For shortWGS, library preparation and paired-end 2 × 100 nt or 2 × 75 nt sequencing with x30 coverage for blood and x90 coverage for tumor DNA were performed at BGI, Copenhagen, Denmark, or at the CMD facility, Department of Clinical Genetics and Pathology, Lund, Sweden. When comparing groups, only one sample per case (the first from each case) was used for calculations.

### Segmentation of bulk DNA copy number data

Tumor Aberration Prediction Suite (TAPS) and Affymetrix Power Tools (https://github.com/rcallahan/affymetrix-power-tools), with an adaptation for the GRCh38/hg38 genome build, were used for segmentation of CN shifts, CN evaluation, and visualization of the SNP array data^36^. CN levels 1, 2, and 3 were assumed to correspond to log values −0.3, 0, and 0.3. For WGS data, ASCAT with a gamma value of 0.7 was used^37^. Segment files were further filtered to include only segments spanning ≥100 kb and, in addition for SNP array data, ≥ 50 probes; any segment that did not meet these criteria was combined with the nearest preceding segment and given the CN value of that segment (Supplementary Data 2). However, unfiltered segment files were used for the CN assessment of *CDK4*, *HMGA2*, and *MDM2*. In Case 2, the CN profile could only be assessed visually. Examples of differences in segmentation and CN calling between SNP array and shortWGS, as well as effects of filtering, can be seen in Supplementary Data 6.

The copy number status was divided into three subgroups: gain (3-4 copies), low-level amplification (5–9 copies), and high-level amplification (≥10 copies). Note that we used integers when delineating contigs and copy number transitions; a copy number estimate of 2.49 was thus called 2 copies and 2.51 as 3 copies. Continuous stretches, uninterrupted by segments exceeding 100 kb with CN levels <3, were defined as CN-gained contigs. When calculating the CN of *CDK4*, *HMGA2*, and *MDM2*, the genomic locations corresponding to the largest transcripts were used for *CDK4* (NM_000075.4; nt 57,747,727-57,752,310), *MDM2* (NM_002392.6; nt 68,808,177-68,845,544), and *HMGA2* (NM_003483.6; nt 65,824,483-65,966,291).

The cut-off levels used here for defining gain, low-level, and high-level amplification are arbitrary. Furthermore, a correct CN assessment of amplified genes is difficult to achieve in WDLS because of the frequent lack of CN losses, which are important for calibrating CN levels. Hence, the algorithms employed to calculate amplitudes from SNP array or shortWGS data cannot with precision distinguish between subclonality and contamination with normal cells. In addition, while different methods (SNP array vs shortWGS) and CN segmentation tools (TAPS vs ASCAT) were in good agreement with regard to start and stop positions of CN shifts, they provided slightly different CN estimates, especially for higher levels of amplification where shortWGS consistently indicated higher CNs (Table 1; Supplementary Data 1 and 6). Finally, scWGS data demonstrate that CN estimates from bulk DNA data are, at best, an approximation of an average of a wide range of CN levels. For calculations below, SNP array data were, when available, used when comparing CN levels among samples and WGS data when comparing locations of SVs with CN states. Filtered segment files for all chromosomes with CN changes in all samples are provided in Supplementary Data 2 and results for all chromosomes are shown in Supplementary Fig. 1.

CN and frequency plots were generated using the R packages CellScape (https://github.com/bernatgel/CopyNumberPlots) and CopyNumberPlots (https://github.com/bernatgel/CopyNumberPlots).

### Global single-cell DNA copy number profiling

To assess intercellular heterogeneity, we performed low-pass WGS on single cell nuclei (scWGS) from six samples from five cases, including two cases from Group A (Cases 1 and 5) and three from Group B (Cases 8, 9, and 20; Table 1; Supplementary Data 1; Fig. 2). Two separate local recurrences (LR) from Case 20 were analyzed: LR2 (sample 20b) and LR3 (sample 20c). Single-cell nuclei were isolated by cell sorting and further processed for sequencing using a Bravo Automated Liquid Handling Platform (Agilent Technologies)^38,39^. Sequencing was performed on an Illumina NextSeq 450 at ERIBA (Illumina). The raw sequencing data were initially demultiplexed using unique barcodes specific to each library and then transformed into fastq format utilizing the Illumina software, bcl2fastq version 1.8.4. The resultant demultiplexed reads were subsequently aligned using Bowtie2 (version 2.2.4), and duplicate reads were flagged and eliminated using BamUtil (version 1.0.3.). Finally, the mapped sequencing reads were subjected to analysis and quality control using AneuFinder (version 1.4.0), which utilized 1 Mb or 40 kb bins for analysis^38^. Only cells with gain or amplification in 12q were kept for further analyses. The number of informative cells, i.e., with CN gain in 12q, ranged from 8–67 per sample (Table 1; Supplementary Data 1; Fig. 2a and b). The heterogeneity score was calculated for each sample, adjusting for patient gender, to assess the extent of copy number heterogeneity among cells^38^. Data presented as heatmaps were generated using AneuFinder.

### Structural variants (SVs)

SVs were detected using shortWGS (as described above in section: Global bulk DNA copy number profiling) and long read WGS (longWGS). ShortWGS data were processed using the nf-core sarek pipeline version 2.6.1 (https://nf-co.re/sarek/2.6.1) including SV calling and annotation^40^. For all underlying tools, default settings were used. ASCAT output from the pipeline (BAF and CN) was further processed using R package ASCAT with a penalty score of 0.7. For shortWGS, SVs from five cases (Cases 1, 5, 7, 16b, and 20c) were obtained. Case 2 could not be processed by the Software. The data were further filtered, excluding all SVs found in the corresponding blood DNA, with a somatic score of <30, classified as imprecise, or with a SV length of <200 nt (Supplementary Data 3).

Two samples (Cases 5 and 21) were selected for longWGS. PacBio libraries were prepared according to Pacbio’s Procedure & Checklist – Preparing HiFi SMRTbell® Libraries using the SMRTbell Express Template Prep Kit 2.0 and size selected using SageElf, according to the same protocol. Each sample was sequenced on two 8 M SMRT cells on the Sequel II instrument, using the Sequel II sequencing plate 2.0, with 30 h movie time and 2 h pre-extension. SVs were detected using the PBSV analysis tool in SMRTLink v10. Because of the lack of corresponding blood samples, the data were further filtered, including only SVs passing the QC (“Passed”), classified as breakend (BND) events, with a total read depth of ≥8, an alternate read depth of ≥5, an alternate ratio of ≥0.1, and a distance of ≥1000 nt between the breakpoints (Supplementary Data 4).

Circosplots were generated with shinyCircos, an R/Shiny web application for interactive creation of circos plots (https://venyao.shinyapps.io/shinyCircos/)^41^.

### Gene expression and fusion genes

RNA for RNA-seq and global gene expression profiling was available from 20 tumors with extra 12q-material in rod-shaped chromosomes (*n* = 7; Group A) or ring chromosomes (*n* = 13; Group B) (Table 1; Supplementary Data 1); the profiles were compared with those in eight *HMGA2* fusion-positive conventional lipomas without 12q-gain and six normal fat samples. RNA extraction, library preparation, and mRNA sequencing of paired-end 150 nt reads were performed as described^42,43^. FusionCatcher with default settings was used to assess fusion transcripts^44^. Filtering criteria are shown in Supplementary Data 5.

For gene expression analysis reads were aligned with STAR (STAR/2.5.0a)^45^. To estimate gene expression levels, RSEM (RSEM/1.2.30) was performed on the aligned data^46^. A count table with tpkm normalized values was used for downstream analysis in Qlucore Omics Explorer 3.8 (Qlucore, Lund, Sweden). Before analysis, the count table was log2 transformed. The expression levels for *CDK4*, *HMGA2*, and *MDM2* were compared with eight lipomas with *HMGA2* fusions and six normal fat samples. The Tukey range test was used as statistical test. The data were variance filtered, selecting a standard deviation of 0.345; an unsupervised hierarchical clustering based on the remaining 1109 genes was generated (Fig. 5).

Quantitative RT-PCR (qRT-PCR) was performed on 22 samples from 20 patients using probes for exons 1-2 and 4-5 of *HMGA2* to compare the relative expression of the 5’- and 3’-ends of *HMGA2*, as described^31^. The following probes: Hs00171569_m1 (*HMGA2* exons 1–2) and Hs00971725_m1 (*HMGA2* exons 4–5) (Thermo Fisher Scientific, Waltham, MA USA) were used in the qRT-PCR. The target value was normalized to *ACTB* (Thermo Fisher Scientific, Waltham, MA USA) as an endogeneous control with Human Adipose Tissue Total RNA (TaKaRa, Shiga, Japan) as the calibrator. Log_10_ values > 5 for exons 1-2 and/or exons 4-5 of *HMGA2* were considered as overexpression. An expression level of exons 1-2 at least 5 times higher than exons 4-5 was defined as a differential expression (Fig. 5; Supplementary Fig. 4).

### Statistics and reproducibility

Statistical tests were performed using the Qlucore Omics Explorer 3.8 (Qlucore, Lund, Sweden) software. The Tukey range test was used as a statistical test for the expression analysis of *CDK4*, *HMGA2* and *MDM2*. The data for the unsupervised heatmap were variance filtered, selecting a standard deviation of 0.345. The heatmap was hierarchically clustered. Sample size: Normal fat (*n* = 6), Lipoma (*n* = 8), rod-shaped (*n* = 7, Cases 1–7), ring (*n* = 13, Cases 8–21).

### Fluorescence in situ hybridization (FISH)

In addition to previously published FISH results^13^, bacterial artificial chromosome (BAC) clones were used to study the location of gained segments in chromosome arm 12q in seven samples from seven patients. The BAC probes were obtained from the BACPAC Resource Center (http:/bacpacresources.org; Supplementary Table 1). The Vysis LSI *MDM2* SpectrumOrange Probe from Abbott Molecular was used to investigate the location and number of copies of the *MDM2* gene. Clone preparation, hybridization, and analysis were performed as described, with minor changes^47^.

### Chromosome banding analysis

Cell culturing and chromosome banding analysis of 21 samples from 17 patients (Table 1; Supplementary Data 1) were performed as described^30^. The nomenclature of the karyotypes followed the guidelines of the International System for Human Cytogenetic Nomenclature^48^.

The GRCh38/hg38 build was used as the human reference genome for all analyses.

### Reporting summary

Further information on research design is available in the Nature Portfolio Reporting Summary linked to this article.

### Supplementary information

Supplementary Information

Description of Supplementary Materials

Supplementary Data 1

Supplementary Data 2

Supplementary Data 3

Supplementary Data 4

Supplementary Data 5

Supplementary Data 6

Supplementary Data 7

Reporting summary

## Data Availability

Segmentation files from SNP array and shortWGS data are presented in the Supplementary Material (Supplementary Data 2). SVs from shortWGS and longWGS are listed in the Supplementary Material (Supplementary Data 3 and 4). RNA-seq, shortWGS, and longWGS data have been deposited at the European Genome-phenome Archive (EGA), which is hosted by the EBI and the CRG, under accession number EGAD50000000087. Further information about EGA can be found on https://ega-archive.org^49^. Numerical source data for the underlying graphs in Fig. 5 can be found in Supplementary Table 2 and Supplementary Data 7. ScWGS data for this study have been deposited in the European Nucleotide Archive (ENA) at EMBL-EBI under accession number PRJEB64351. Any additional information is available upon request.
