# Supplementary material for: MDM2 amplification in rod-shaped chromosomes provides clues to early stages of circularized gene amplification in liposarcoma
Source: Commun Biol. 2024 May 20;7:606. doi: 10.1038/s42003-024-06307-1 (PMC11106292; doi:10.1038/s42003-024-06307-1)
Supplement: Supplementary file 1 — Supplementary Information [file 42003_2024_6307_MOESM1_ESM.pdf]

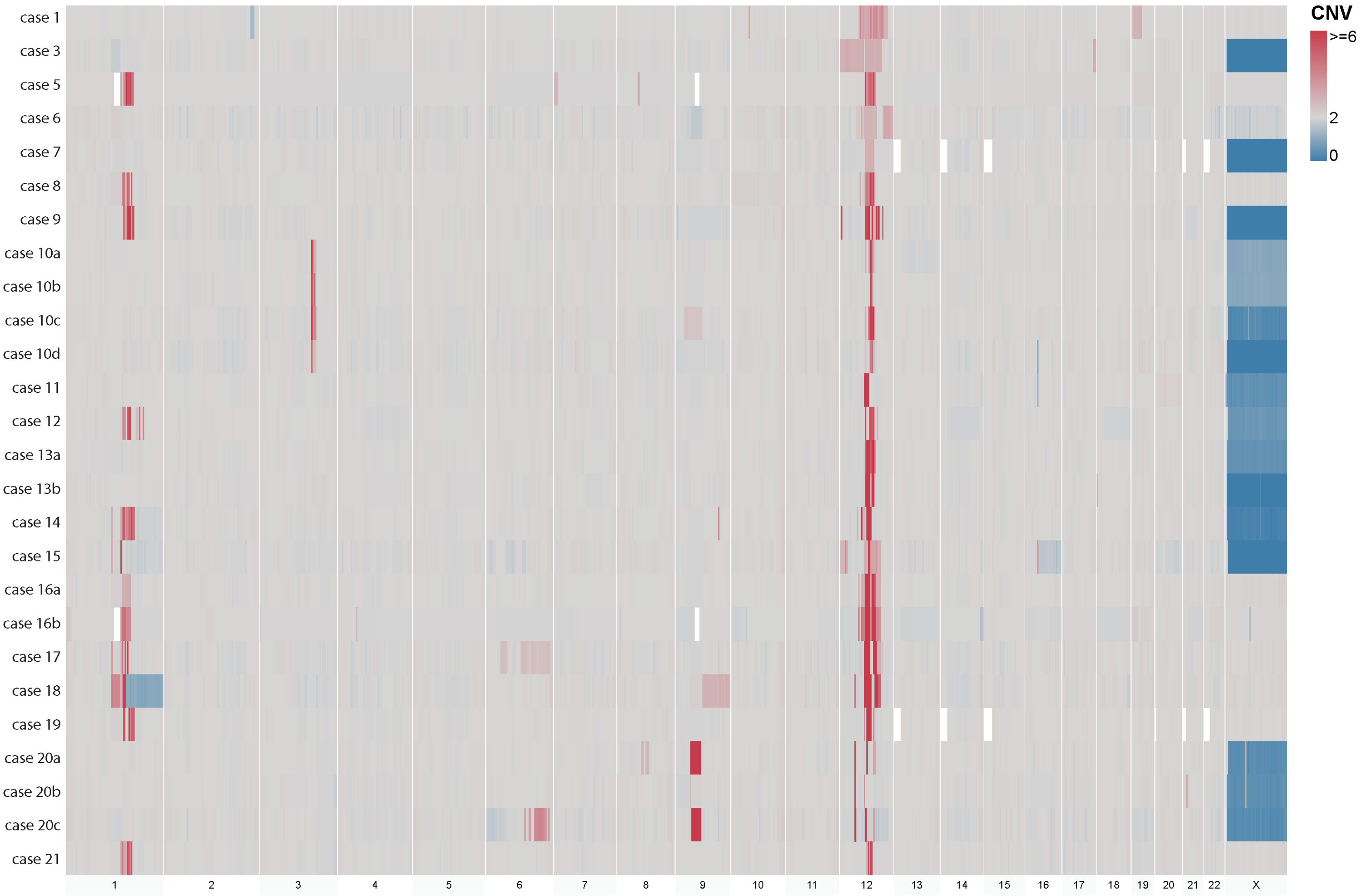

Supplementary Fig. 1. Copy number (CN) variation in lipomatous tumors with 12q gain. Distribution of genome-wide CN changes as detected in tumor biopsies with SNP array or short-read whole-genome sequencing.

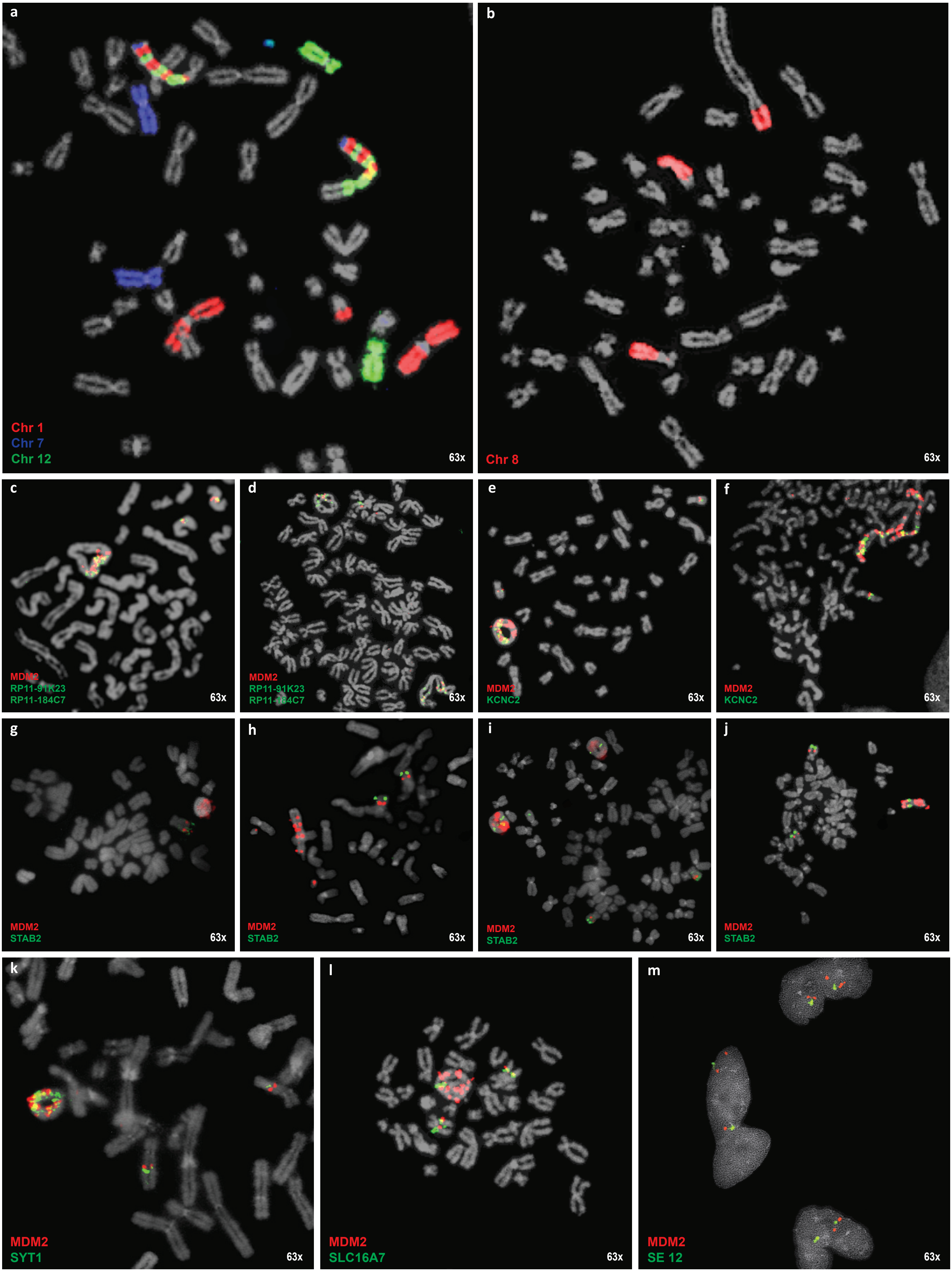

Supplementary Fig. 2. Fluorescence in situ hybridization (FISH). FISH was performed on metaphase spreads (a;b) to confirm joining of chromosomes 1, 7, 8, and 12 in Case 5, to show the interchangeable nature of ring chromosomes and marker chromosomes in Cases (c;d) 8, (e;f) 10c, (g;h) 17, and (i;j) 18, to evaluate the location of low-level gained sequences (green signals; MDM2 in red) from 12q in Cases (c;d) 8, (e;f) 10c, (k) 13a, (l) 14, and (i;j) 18, and (m) on interphase nuclei to confirm gain of only one copy of MDM2 in Case 7.

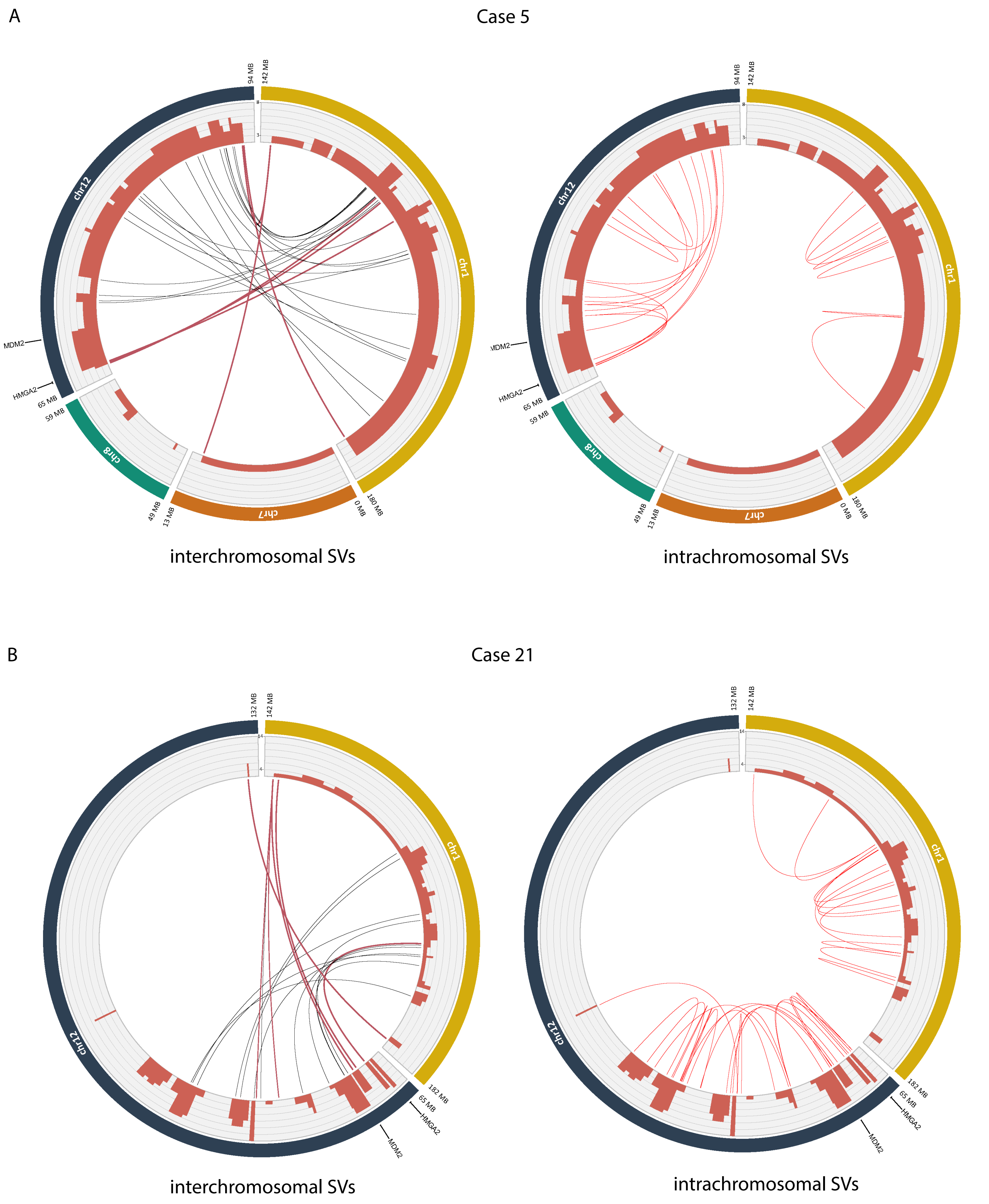

Supplementary Fig. 3. Inter- (left) and intrachromosomal (right) structural variants (SVs) detected at long-read whole genome sequencing in lipomatous tumors with 12q-gain.

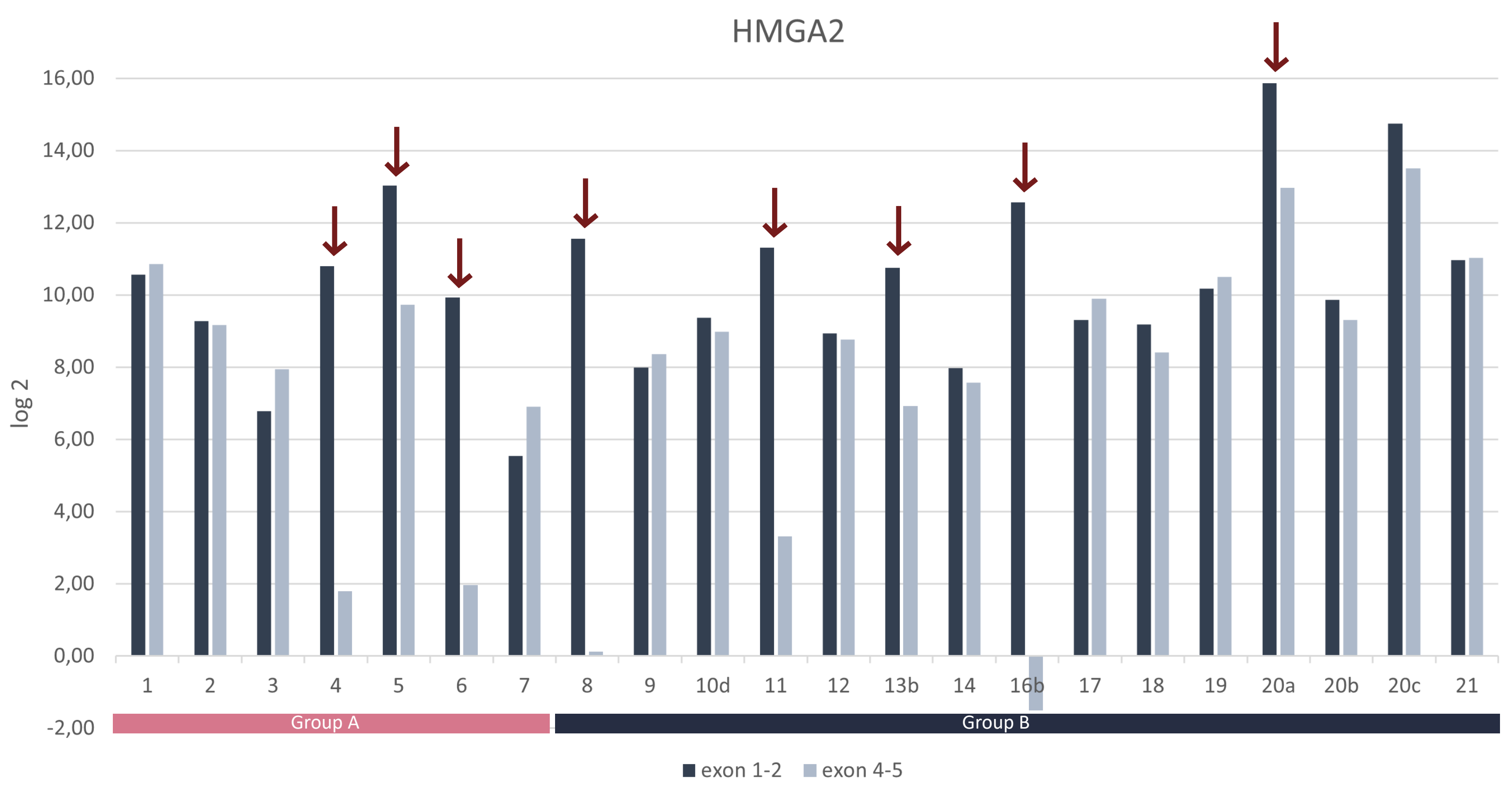

Supplementary Fig. 4. Expression levels of 5'-HMGA2 (exons 1-2) and 3'-HMGA2 (exons 4-5) at qRT-PCR. Dark red arrows indicate differentially expressed exons (exons 1-2 vs exons 4-5).

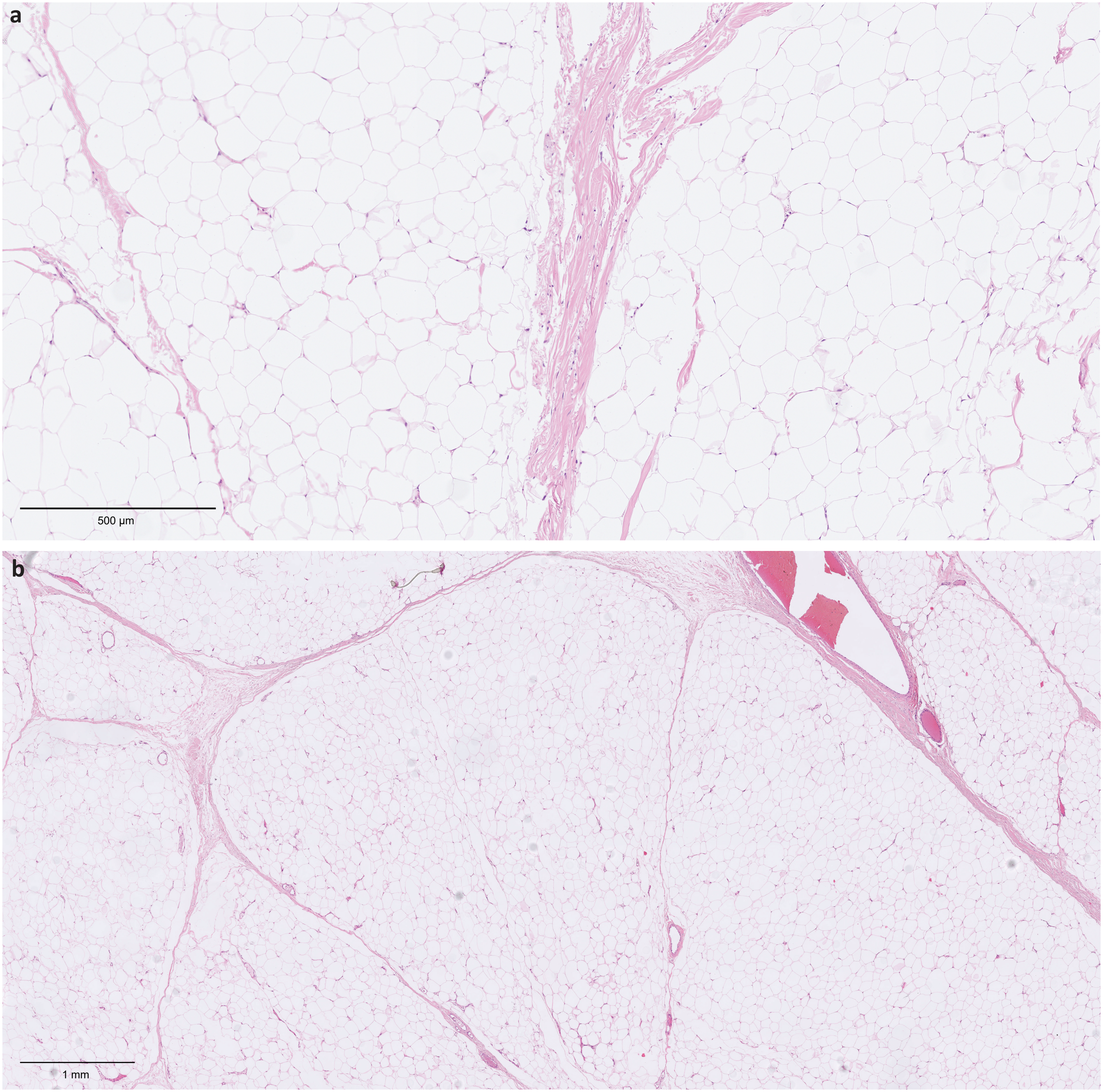

Supplementary Fig. 5. Morphological images (hematoxylin-eosin stain) showing lipoma-like morphology in tumors with 12q-gain: (a) Case 7, diagnosed as WDLS, with gain of one extra copy of MDM2 in rod-shaped chromosome and (b) Case 9, diagnosed as WDLS with minimal atypia, with gain of four extra copies of MDM2 in ring chromosomes. Both tumors showed a lobular architecture and consisted of mature adipocytes of varying size. Overall, the tumors showed a lipo-

Supplementary Table 1: Bacterial artifical chromosome (BAC) clones used for FISH analysis.

Case	BAC-name	Gene	Nt position according to hg19 or hg38
**8**	RP11-184C7	Chr12: 66.7 - 68.8 MB	chr12:66803069-66966970 (hg38)
	RP11-91K23	Chr12: 66.7 - 68.8 MB	chr12:67118349-67284068 (hg38)
	Vysis LSI MDM2	MDM2	chr12q15
**9**	RP11-984M1	EEA1	chr12:92881999 -93063324 (hg19)
	RP11-756G20	EEA1	chr12:92673823-92868401 (hg19)
	Vysis LSI MDM2	MDM2	chr12q15
**10c**	RP11-910G16	KCNC2	chr12:75319216-75519610 (hg19)
	RP11-963J20	KCNC2	chr12:75563921-75774869 (hg19)
	Vysis LSI MDM2	MDM2	chr12q15
**13a**	RP11-90C1	SYT1	chr12:78695292-78883159 (hg19)
	RP11-230I13	SYT1	chr12:79773553-79954229 (hg19)
	RP11-530C5	SYT1	chr12:80044061-80221794 (hg19)
	Vysis LSI MDM2	MDM2	chr12q15
**14**	RP11-597J22	SLC16A7	chr12: 59958470-60119978 (hg19)
	RP11-383F9	SLC16A7	chr12: 60333395 - 60507033 (hg19)
	RP11-609E19	SLC16A7	chr12:60569418-60714840 (hg19)
	RP11-26N11	SLC16A7	chr12:60974515-61130275 (hg19)
	Vysis LSI MDM2	MDM2	chr12q15
**17 and 18**	RP11-205I24	STAB2	chr12:103572435-103729787 (hg38)
	RP11-1102P10	STAB2	chr12:103403468-103568244 (hg38)
	RP11-146C22	STAB2	chr12:103789132-103974508 (hg38)
	Vysis LSI MDM2	MDM2	chr12q15

Supplementary Table 2: Numerical source data for the *HMGA2* qRT-PCR analysis.

- RQ data are log2 transformed

Case	*HMGA2* ex 1-2	*HMGA2* ex 4-5
1	10,56373134	10,86270487
2	9,268179385	9,168521542
3	6,774918807	7,947473301
4	10,79626631	1,793841171
5	13,02195813	9,733249318
6	9,932565004	1,959937576
7	5,547619918	6,909610437
8	11,55489338	0,124328135
9	7,983706193	8,36610798
10d	9,369403229	8,983808781
11	11,31392207	3,315421316
12	8,935986575	8,769890171
13b	10,74597846	6,921959917
14	7,970819151	7,568852967
16b	12,56225101	-1,514573173
17	9,301398666	9,899345723
18	9,189061013	8,401178188
19	10,17741954	10,49984589
20a	15,86667157	12,96280294
20b	9,858459741	9,308889607
20c	14,74353317	13,50968174
21	10,96236271	11,02880128
