# Supplementary material for: MDM2 amplification in rod-shaped chromosomes provides clues to early stages of circularized gene amplification in liposarcoma
Source: Commun Biol. 2024 May 20;7:606. doi: 10.1038/s42003-024-06307-1 (PMC11106292; doi:10.1038/s42003-024-06307-1)
Supplement: Supplementary file 2 — Description of Supplementary Materials [file 42003_2024_6307_MOESM2_ESM.docx]

**Description of Additional Supplementary Files**

**File name:** Supplementary Data 1

**Description:** General data about the tumor samples

**File name:** Supplementary Data 2

**Description:** Filtered copy number segment data

**File name:** Supplementary Data 3

**Description:** Structural variant data from whole genome Sequencing

**File name:** Supplementary Data 4

**Description**: Structural variant data from long read whole genome sequencing

**File name:** Supplementary Data 5

**Description:** Filtered fusion transcripts

**File name:** Supplementary Data 6

**Description:** Differences between SNP array and whole genome sequencing with regard to copy number segmentation and effects of filtering

**File name:** Supplementary Data 7

**Description:** RNAseq numerical source data
